# Altered neural responses to social fairness in bipolar disorder

**DOI:** 10.1016/j.nicl.2020.102487

**Published:** 2020-11-03

**Authors:** Giannis Lois, Eva E. Schneider, Aleksandra Kaurin, Michèle Wessa

**Affiliations:** a Department of Clinical Psychology and Neuropsychology, Institute of Psychology, Johannes Gutenberg-University Mainz, Germany; b Department of Microeconomics and Public Economics, School of Business and Economics, Maastricht University, The Netherlands; c German Resilience Center, Mainz, Germany; d Department of Psychology, University of Pittsburgh, USA

**Keywords:** Bipolar disorder, Social decision-making, Ultimatum game, Fairness, Ambiguity

## Abstract

Bipolar Disorder (BD) has a debilitating impact on psychosocial functioning and social decision-making. Recent evidence using the Ultimatum Game (UG) has shown increased rejection of moderately unfair offers in BD, suggesting impaired processing of ambiguous social information related to fairness. The present study builds upon this finding to investigate the neural substrates of fairness processing in BD. During functional magnetic resonance imaging scanning, euthymic BD patients (n = 41) and matched healthy controls (HC; n = 41) accepted or rejected very unfair, moderately unfair, or fair offers in the UG. Acceptance rates of moderately unfair offers were significantly lower in BD patients. This aberrant behavior co-occurred with abnormal brain responses to moderately unfair offers. Compared to HC, BD patients exhibited hypoactivation of right anterior insula in response to moderately unfair offers suggesting impaired integration of affective and contextual information. BD patients also displayed stronger deactivation of posterior and middle insula in response to moderately unfair offers reflecting impaired processing of the contextual aspects of fairness. The level of impulsivity of BD patients positively correlated with the abnormal deactivation of posterior and middle insula. A separate analysis revealed increased activation of dorsal ACC and left ventrolateral PFC in response to rejected compared to accepted offers in BD patients. Taken together, our findings suggest impaired processing of ambiguous social information in euthymic BD patients which is associated with increased rejection of moderately unfair offers. This impairment may reflect a failure to integrate contextual information and may be related to increased trait impulsivity.

## Highlights

• Bipolar disorder is characterized by impaired processing of social fairness.
• BD patients exhibit increased rejection of moderate unfairness in Ultimatum Game.
• BD patients display decreased response to moderate unfairness in anterior insula.
• BD patients deactivate posterior and middle insula in response to unfairness.
• Trait impulsivity positively correlated with deactivations in posterior insula.

## 1 Introduction

The impact of mood disorders, such as Bipolar Disorder (BD), on social decisions has been - to a large extent- neglected. BD is characterized by deficits in psychosocial functioning (Goswami and Moore, 2006, Ibanez, 2012). After the manifestation of the disorder, the frequency and quality of social activities decrease dramatically in BD patients (Grover et al., 2017, Pope et al., 2007, Tatay-Manteiga et al., 2018). Some of these impairments in social behavior, persist even after remission and have dramatic consequences for the quality of life of BD patients (Rossell and Rheenen, 2014). In an effort to examine the interpersonal dysfunctions that characterize BD, the present study sets out to investigate the neurobehavioral substrates of social decision-making in BD patients.

A well-established way to investigate decision-making in interpersonal contexts is the use of social economic game paradigms (Guth and Kocher, 2014). One of the most extensively studied social decision-making paradigm is the Ultimatum Game (UG). This economic game has provided insights into the behavioral and neural underpinnings of processing social fairness (Guth and Kocher, 2014, Feng et al., 2015). In the UG, one player, the proposer, offers a split of money to the actual participant, the responder, who then decides whether to accept the offer or reject it, in which case both players receive nothing. From a rational economic perspective, the optimal choice for the responder is to accept any offer, as any monetary amount is preferable to none. However, it is well replicated that healthy subjects are willing to reject low offers (<20–30% of the total amount) in order to punish unfair proposers and enforce the fairness norm (Fehr and Schmidt, 1999).

Imaging studies on healthy subjects have reported that brain regions related to processing aversive emotional information (i.e. anterior insula), cognitive conflict (i.e. dorsal anterior cingulate cortex) and cognitive control (i.e. dorsolateral prefrontal cortex) are activated in response to unfair offers (Sanfey et al., 2003). By contrast, fair offers activate reward-linked brain regions such as the ventral striatum and the ventromedial prefrontal cortex (vmPFC) (Tabibnia and Lieberman, 2007) and posterior and middle insular regions consistent with their role in coding fairness and equality (Hsu et al., 2008, Wright et al., 2011).

In the last decade, the UG has been employed to study social decision-making in patients with diverse mental disorders such as depression, schizophrenia, and others (Hinterbuchinger et al., 2018). However, to date, only one study has examined the behavioral responses of BD euthymic patients in the UG (Duek et al., 2014). While this study found similar acceptance rates of fair (50% of total amount) or very unfair (<25% of total amount) offers among euthymic BD patients and healthy controls (HC), BD patients were more likely to reject moderately unfair offers (i.e. offers between 30 and 40% that are usually perceived as fair) (Duek et al., 2014).

Evidence from healthy individuals suggests that fair and very unfair offers elicit strong behavioral tendencies that minimize individual differences at the behavioral level (Guth and Kocher, 2014, Ferguson et al., 2014). On the other hand, moderately unfair offers are processed longer and responses to these offers are more sensitive to contextual changes (Ferguson et al., 2014). These offers most likely require higher attentional and cognitive control to respond, as one has to integrate contextual information (e.g. proposer’s available alternative options, intentions, prior experiences with offers, etc.) to decide the appropriate course of action. In this respect, it can be argued that the low acceptance rate of moderately unfair offers in BD reflects cognitive control deficits (Kanske et al., 2015, McTeague et al., 2017) and higher trait impulsivity (Etain et al., 2013) that characterize this clinical population. This view is consistent with the finding that BD patients experienced higher levels of anger in response to unfair offers while some also expressed regret at behaving impulsively and rejecting offers that lost them profit (Duek et al., 2014).

In the current study, we used functional magnetic resonance imaging (fMRI) to investigate, for the first time, neural responses to social fairness in euthymic BD patients. The main aim of this study is to obtain a more comprehensive picture of the underlying cognitive and neural processes that underlie the reported behavioral abnormalities (Duek et al., 2014) by separately examining the neural responses to very unfair, moderately unfair, and fair UG offers. We hypothesized that BD patients would demonstrate reduced recruitment of brain regions that are implicated in the cognitive processing of fairness (i.e. anterior insula and dorsal ACC) and are important for the processing of the contextual aspects of fairness (i.e. posterior and middle insular regions). Furthermore, we explored whether these hypothesized abnormal brain responses to fairness are specific to moderately unfair offers. Given that euthymic BD patients expressed increased anger during the UG and reported regret at impulsively rejecting unfair offers (Duek et al., 2014), we also examined the brain activation pattern when BD patients reject (compared to accept) unfair offers. Lastly, we explored whether individual differences in hypersensitivity to emotional stimuli and elevated trait impulsivity that characterize BD, predict abnormal behavioral and brain responses to unfair offers.

## 2 Materials and methods

### 2.1 Participants

Euthymic BD-I patients were recruited at the Outpatient Clinic of the University of Mainz, through local psychiatrists, and through press releases. Diagnoses of BD and potential comorbid mental disorders were assessed with the German version of the Structured Clinical Interview for DSM-IV, SCID-I and –II (First et al., 1995) and were conducted by trained clinical psychologists. None of the patients fulfilled the criteria for any other current mental disorder. The medication status (i.e. type and dose) of all patients had been stable during the previous 6 months. Total and type‐specific medication load was calculated according to an algorithm based on the dose and the type of medication (Sackeim, 2001, Phillips et al., 2008).

Healthy participants were recruited through the registry office of the city of Mainz and advertisement in public facilities. Healthy participants were also assessed with the SCID-I and -II to confirm that they are free of any past or present mental disorders. Exclusion criteria for all participants were, age < 18 or > 65, neurological disorder or head trauma with unconsciousness, presence of substance abuse or dependence for at least 3 months prior to testing, and common MRI exclusion criteria. Variables describing the clinical course of the disease such as the number of past depressive and manic episodes, the age at illness onset, and the time in remission were acquired for every patient (Table 1). These clinical variables and the total and type-specific medication load were also included as covariates in an exploratory regression analysis for both behavioral responses and brain activation.

**Table 1:** Sample characteristics.

Characteristics	HC (n = 41)		BD (n = 41)		Statistics	p-value
	N	%	N	%		
Female	21	51.2	21	51.2		
	Mean	SD (Range)	Mean	SD (Range)		
Age	45.3	13.8 (First et al., 1995, Sackeim, 2001, Phillips et al., 2008, Patton et al., 1995, Larsen et al., 1987, Hamilton, 1960, Young et al., 1978, Gabay et al., 2014, Harle and Sanfey, 2007, Feinberg et al., 2010, Woo et al., 2014, Corradi-Dell'Acqua et al., 2013, Botvinick et al., 2004, Civai et al., 2012, Xiang et al., 2013, Zhou et al., 2014, Sanfey and Chang, 2008, Grecucci et al., 2013, Harle et al., 2012, Chang et al., 2013, Kelly et al., 2012, Janiri et al., 2019, Swann et al., 2003, Baez et al., 2013, Brambilla et al., 2007)	44.6	13.4 (First et al., 1995, Sackeim, 2001, Phillips et al., 2008, Patton et al., 1995, Larsen et al., 1987, Hamilton, 1960, Young et al., 1978, Gabay et al., 2014, Harle and Sanfey, 2007, Feinberg et al., 2010, Woo et al., 2014, Corradi-Dell'Acqua et al., 2013, Botvinick et al., 2004, Civai et al., 2012, Xiang et al., 2013, Zhou et al., 2014, Sanfey and Chang, 2008, Grecucci et al., 2013, Harle et al., 2012, Chang et al., 2013, Kelly et al., 2012, Janiri et al., 2019, Swann et al., 2003, Baez et al., 2013, Brambilla et al., 2007)	t(80) = -0.25	p = 0.80
Formal education, years	16.8	1.9 (Wright et al., 2011, Hinterbuchinger et al., 2018, Duek et al., 2014, Ferguson et al., 2014, Kanske et al., 2015, McTeague et al., 2017, Etain et al., 2013, First et al., 1995, Sackeim, 2001)	15.5	2.3 (Sanfey et al., 2003, Tabibnia and Lieberman, 2007, Hsu et al., 2008, Wright et al., 2011, Hinterbuchinger et al., 2018, Duek et al., 2014, Ferguson et al., 2014, Kanske et al., 2015, McTeague et al., 2017, Etain et al., 2013, First et al., 1995, Sackeim, 2001)	t(80) = -2.73	p = 0.008
Income	3726	2562 (500–11000)	2727	3440 (404–18000)	t(78) = 1.47	p = 0.146

Current symptoms						
HAM-D	0.5	0.9 (0–4)	1.5	1.8 (0–7)	t(75) = 2.91	p = 0.005
YMRS	0.2	0.6 (0–2)	1.2	1.9 (0–6)	t(74) = 2.85	p = 0.006

Clinical characteristics						
No. of depressive episodes	–		6.9	6 (1–30)		
No of manic episodes	–		5	8.1 (1–50)		
Age at illness onset (years)	–		25.2	10.4 (7–51)		
Time in remission (months)	–		34.9	41.5 (2–194)		
Total med load	–		2.1	1.2 (0–4)		
SSRI antidepressants med load	–		0.3	0.7 (0–2)		
Other antidepressants med load	–		0.2	0.6 (0–2)		
Mood stabilizers med load	–		0.8	0.9 (0–2)		
Antipsychotics med load			0.5	0.8 (0–2)		

Personality traits						
BIS11	56.2	7.7 (38–71)	64.3	10.2 (47–87)	t(80) = 4.07	p < 0.001
AIM	20.4	14.2 (-6–51)	33.1	19.7 (-7–75)	t(80) = 3.36	p = 0.001

Fifty euthymic BD-I patients and 50 healthy controls matched for age (+/- 2 years), and gender participated in the study. Nine patients were excluded from the analysis due to missing data (Tatay-Manteiga et al., 2018) excessive movement (Goswami and Moore, 2006), and misdiagnosis (Grover et al., 2017). As healthy controls were matched on an individual basis, the nine healthy controls were also excluded resulting in a final sample of 41 BD patients and 41 healthy controls. All participants gave their written informed consent to participate in the study. The study was approved by the ethics committee at the Psychological Institute of the University in Mainz and adhered to the Declaration of Helsinki.

### 2.2 Questionnaires

Trait impulsivity was assessed using the Barratt Impulsiveness Scale (BIS-11) (Patton et al., 1995). This questionnaire consists of 30 items and yields three second-order factors (i.e. attentional, motor, and non-planning impulsiveness). The strength or weakness with which one experiences emotions was measured by the Affect Intensity Measure (AIM) (Larsen et al., 1987). AIM is a 29-item questionnaire that consists of three sub-scales (i.e. positive affectivity, negative reactivity, and lack of serenity). Overall, internal consistency was good for both questionnaires (for BIS-11 α = 0.83 and for AIM α = 0.80). Residual depressive and manic symptoms were assessed by the Hamilton Depression Rating Scale (HAMD) (Hamilton, 1960) and the Young Mania Rating Scale (YMRS; (Young et al., 1978) respectively. Remission was defined as a score < 7 on the HAM-D and the YMRS for at least 8 weeks.

### 2.3 Experimental paradigm and procedure

Before scanning, participants were instructed that while in the scanner they would be presented with monetary offers made by other players (each player making only one offer) with regard to a split of a given amount of money (i.e. 10€). Participants performed the UG as responders inside the scanner and had to choose whether to accept or reject monetary offers made by other players. By accepting the offer, the money would be split as proposed. In the case of rejection, both players would receive nothing on that trial. Participants were instructed that the offers had been previously made by other proposers and added to the paradigm. To ensure that participants would not raise suspicions about the existence of the proposers, they were told that they would also play in the role of proposer and made one offer. This offer would be presented to another participant in a future session. In reality, the offers were preprogrammed. This deception was necessary to deliver a controlled task to each participant while ensuring the ecological validity of the task. Participants were informed that apart from the participation fee, they would receive compensation proportional to the amount of money earned during the experiment. In reality, all participants earned a fixed amount of 5€ as earnings based on their decisions in the Game. At the end of the experimental session, an informal survey was administered to assess whether participants believed that offers were genuinely human. None of the participants expressed doubts regarding the cover story. Participants were completely debriefed about the deception and the aim of the study at the end of the experimental session.

Each experimental session comprised 40 randomized trials. Participants received offers that ranged from 1, 2, 3, 4 or 5€ out of 10€. Behavioral studies have reliably shown that typically offers below the 25% of the total amount are rejected (70–90% rejection rate) while offers of 25–40% of the total amount are predominantly accepted (0–35% rejection rate) (Gabay et al., 2014). For this reason, in line with previous studies in healthy individuals (Ferguson et al., 2014, Harle and Sanfey, 2007), 9–1 and 8–2 offers were defined as very unfair, 7–3 and 6–4 offers were defined as moderately unfair, and 5–5 offers were defined as fair. Importantly, this categorization is consistent with previous evidence showing an abnormal behavioral response of BD patients only to moderately unfair offers (Duek et al., 2014). In total, participants received 16 very unfair trials, 14 moderately unfair trials, and 10 fair trials. The sequence of trial types and inter-trial timing variation (‘jitter’) was determined using the Optseq (http://surfer.nmr.mgh.harvard.edu/optseq/) algorithm, designed to optimize detection of the neural signals of interest.

Participants lay supine in the MR scanner with their head fixated. Stimuli were presented using Presentation 11.0 (Neurobehavioral Systems). On each trial, participants were presented with the name of a co-player for 1 sec. Subsequently, participants are presented with the co-player's offer. After 4 sec, participants are given up to 3 sec to accept or reject the offer. Trials in which participants failed to respond on time (<2% of total trials) were excluded from the analysis. After their response, participants receive feedback about their and the co-player’s outcome (1.5 sec). Trials were followed by a jittered inter-trial interval ranging from 2.9 to 6.1 sec.

### 2.4 Behavioral analysis

Acceptance rates were compared between the groups and across the different levels of unfairness by performing a mixed ANOVA test with the two groups (HC and BD) as between-subject factor and the three levels of unfairness (very unfair, moderately unfair, and fair) as within-subject factor. To account for potential confounding effects of sociodemographic variables, we performed additional analyses including age, gender, years of education, and income as covariates. Due to the positively skewed distribution of reaction times (RTs), we fitted a generalized linear mixed model (with subjects as a random factor) using a gamma density link function into the RT data. Moreover, post-hoc tests were used to examine whether the expected increase in RTs in moderately unfair offers is present in both groups.

### 2.5 MRI data acquisition

MRI data were acquired on a 3 Tesla MR scanner (MAGNETOM trio; Siemens) by using a 32-channel head coil. A multiband echoplanar imaging (EPI) sequence (TR = 1000 ms, TE = 29 ms, flip angle = 56°, FOV = 210 mm, voxel size = 2.5 × 2.5 × 2.5 mm3, 60 slices, MB acceleration factor = 4) was used for blood oxygen-level dependent (BOLD) fMRI to achieve a fine-grained temporal resolution (Feinberg et al., 2010). Two sessions of functional images were acquired, each containing 300 brain volumes. A high-resolution T1-weighted structural image using the MPRAGE sequence (TR: 1.9 s; TE: 2.52 ms; flip angle = 9°; 1 × 1 × 1 mm isotropic voxels) was also acquired for normalization.

### 2.6 MRI data analysis

MRI data were analyzed in SPM12 (http://www.fil.ion.ucl.ac.uk/spm/). To establish equilibrium magnetization., the first four EPI images of each run were discarded. Image preprocessing included spatial realignment to the first image, co-registration of the T1-weighted anatomical image to the mean functional image, normalization to the MNI template via segmentation, and spatial smoothing with an 8 mm FWHM Gaussian kernel. Subjects with excessive movement (translations of 2 mm or rotations of 2°) were discarded from subsequent analysis.

At the first level, a general linear model (GLM) was fitted for each subject to model the individual BOLD signal changes induced by the three experimental conditions (i.e. three regressors, one for each level of fairness). An event-related design was implemented with the onset of the offer modelled as boxcar function corresponding in length to the duration of the offer presentation. In a separate GLM analysis, we distinguished between accepted and rejected offers (i.e. two regressors). In this analysis, we modeled the onset of the text prompting a response using a stick function.

For both GLM analyses, regressors of interest were convolved with the haemodynamic response function (HRF) without time or dispersion derivatives. Six head motion realignment parameter estimates were included as covariates of no interest. Low-frequency signal drifts were filtered using a cutoff period of 128 s. Random-effects analyses were performed at the second level using single-subject contrast images of interest to examine within- and between-group activations using one-sample and two-sample t-tests. To examine brain activation in response to very unfair and moderately unfair offers, we contrasted these two types of offers with fair offers.

Hypothesis-driven small volume analyses were performed in a set of predefined regions of interest (ROIs) that have shown abnormal activity in BD and have been reliably implicated in processing unfairness (Feng et al., 2015, Gabay et al., 2014). ROIs were based on peak MNI-coordinates retrieved from a recent *meta*-analysis of UG studies (Feng et al., 2015). 10 mm spheres were created centered on peak MNI-coordinates in bilateral insula (anterior, middle and posterior) and adjacent superior temporal gyrus, dorsal ACC, bilateral ventrolateral and dorsolateral PFC, ventromedial PFC, ventral striatum, and pre supplementary motor area (pre-SMA).

Within this set of ROIs, correction for multiple comparisons was performed using cluster-extent based thresholding (Woo et al., 2014). First, at the voxel level, an uncorrected inclusion threshold of p_unc_ < 0.005 was used to define the suprathreshold voxels. Second, a cluster-level extent threshold was determined using Monte Carlo simulations under the null assumption of no activation in any voxel in that cluster (Woo et al., 2014). This simulation assumes a type I error voxel activation based on the voxel threshold, smoothes the volume with a Gaussian kernel, then counts the number of voxel clusters of a given size. After running a number of iterations, the algorithm calculates a probability associated with each cluster extent, and the cluster extent threshold that yields the desired correction for multiple comparisons can be chosen (Woo et al., 2014). A minimum cluster size of 23 voxels survives a cluster-extent based threshold for a p < 0.05 family-wise error correction. Additional analyses were conducted using age, gender, years of education, and income as covariates to identify potential confounding effects of sociodemographic variables on brain activation. Results from a whole brain analysis are provided in Table S1 of the Supplementary Material.

### 2.7 Correlation analysis

We performed regression analysis to examine the relationship between brain responses at the voxel level and impulsivity, assessed by the BIS11 questionnaire or affect intensity, assessed by the AIM questionnaire. We examined these relationships within the defined set of ROIs. An uncorrected inclusion threshold of punc < 0.001 and a minimum cluster size of 20 voxels were used to define the cluster-extent based threshold for a p < 0.05 family-wise error correction.

We also examined whether group differences in brain activation can be mediated by factors that in general and in this specific sample differ between BD patients and healthy individuals (e.g. impulsivity and affect intensity). We thus performed a mediation analysis by computing indirect effects of group on brain activation through impulsivity and affect intensity using bias-corrected bootstrapping with 10,000 resamples and a 95% confidence interval. Following procedures outlined by Hayes and Preacher (2014), group was dummy-coded.

We also performed Pearson’s correlation analysis within the BD group to examine the relationship between abnormal brain responses and clinical variables, depressive and manic residual symptoms (HAM-D and YMRS), or total and type-specific medication load. Correlations were considered significant if they survived FDR correction for multiple testing at p < 0.05.

## 3 Results

### 3.1 Descriptive statistics

The sociodemographic and clinical characteristics of the two groups of participants are shown in Table 1. Compared to HC, euthymic BD patients had less years of education and exhibited higher BIS11, AIM, HAM-D, and YMRS scores (Table 1). However, YMRS and HAM-D scores were still low in BD individuals and well represented a euthymic state.

### 3.2 Behavioral analysis

**Fig. 1:**
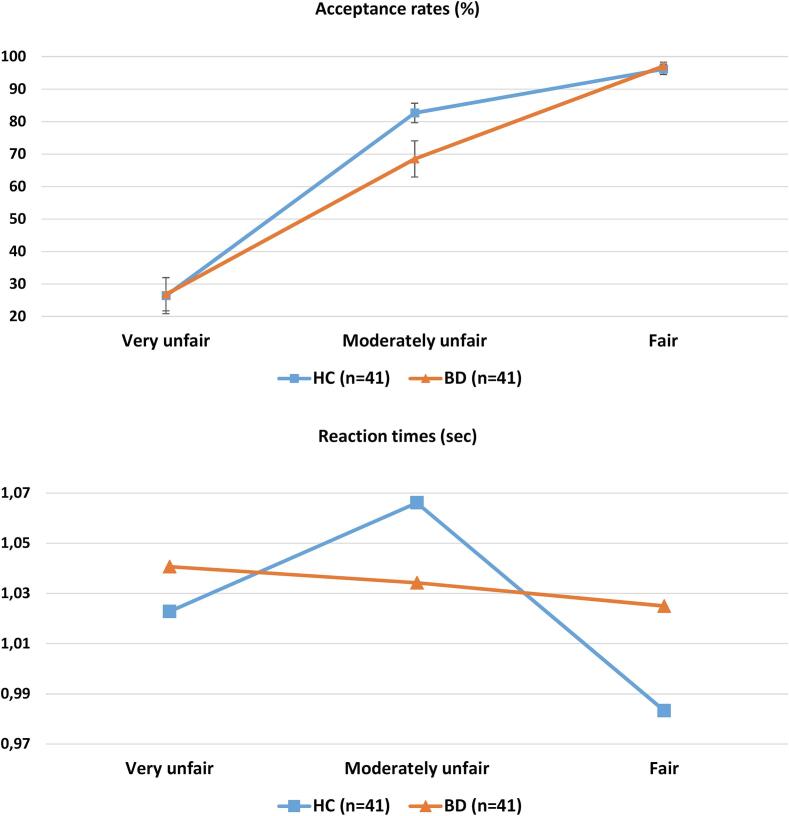
Acceptance rates and reaction times in the UG. A: Percentage of offers accepted by HC (blue) vs BD (orange) in three different levels of fairness. B: RTs (sec) of HC (blue) vs BD (orange) in three different levels of fairness. Error bars represent standard errors. (For interpretation of the references to colour in this figure legend, the reader is referred to the web version of this article.)

As Fig. 1A depicts, acceptance rates increased with increasing levels of fairness in both groups. A 3(fairness) × 2(groups) mixed ANOVA test identified a significant main effect of fairness on acceptance rates (*F*(2,160) = 199.3, *p* < 0.0001, *η_p_^2^* = 0.714). The fairness × group interaction effect was marginally non-significant (*F*(2,160) = 2.55, *p* = 0.061, *η_p_^2^* = 0.034). While acceptance rates in very unfair and fair offers did not differ between the two groups, BD patients displayed lower acceptance rates in response to moderately unfair offers (*t*(80) = 2.23, *p* = 0.029, *d* = 0.49). Controlling for sociodemographic variables (i.e. age, gender, education, and income) did not influence the observed effects on behavior.

The generalized linear mixed model revealed a significant main effect of fairness on (*F*(2, 3215) = 3.10, *p* = 0.045). The fairness × group interaction effect was non-significant *(F*(2, 3215) = 2.03, *p* = 0.131). Given that behavioral aberrations in the BD group were manifested only in moderately unfair offers, separate analysis was conducted to identify effects specific to this type of offers. For this purpose, moderately unfair offers were directly compared with the other two types of offers (i.e. collapsing very unfair and fair offers). This generalized linear mixed model revealed a significant main effect of fairness (*F*(1, 3217) = 4.77, *p* = 0.029) and a marginally significant group by fairness interaction effect (*F*(1, 3217) = 3.87, *p* = 0.049). Taken together, these findings indicate that only the HC group (and not the BD group) displayed longer RTs in response to moderately unfair compared to very unfair or fair offers (Fig. 1B).

### 3.3 Neuroimaging analysis

**Fig. 2:**
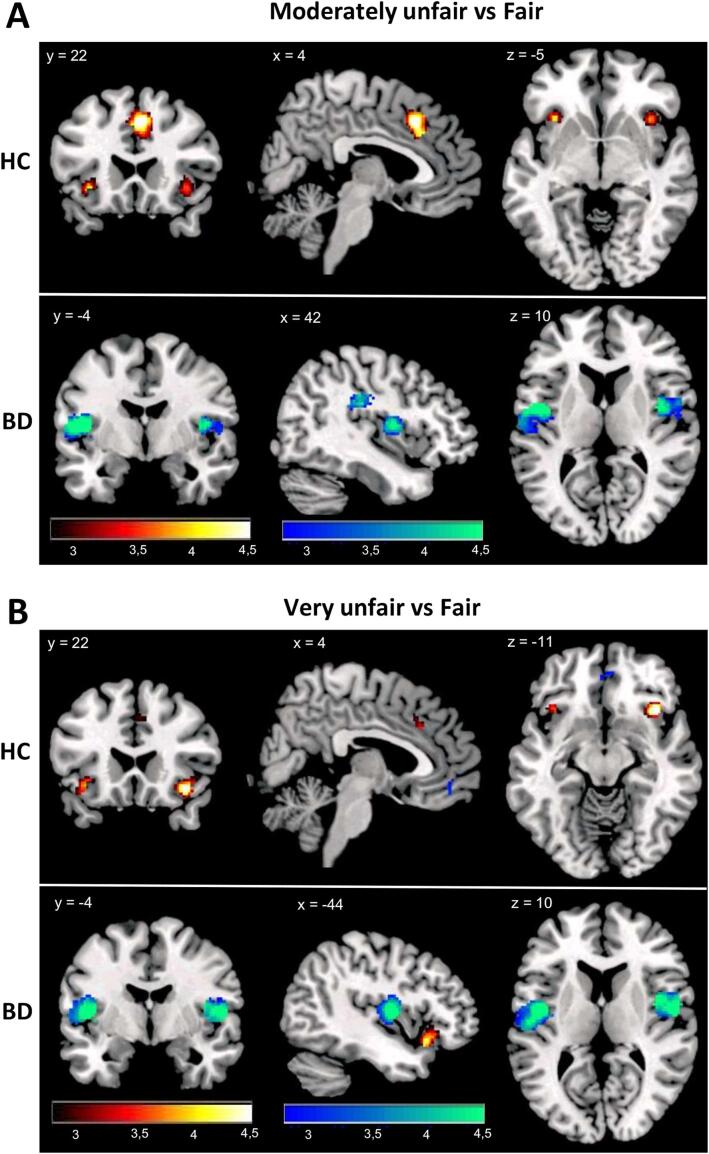
Within-group neural responses to moderately unfair (A) and very unfair (B) offers for HC (upper part of A and B) and BD (lower part of A and B). Activation in response to unfairness is depicted in the red/yellow scale while deactivation in response to unfairness is depicted in the blue/green scale. Images displayed at p < 0.005 initial threshold with a cluster extent threshold of 23 voxels. (For interpretation of the references to colour in this figure legend, the reader is referred to the web version of this article.)

The one-sample t-tests revealed distinct group-specific activation patterns for the contrasts very unfair > fair and moderately unfair > fair. For both contrasts, we observed increased activation in the left anterior insula/left ventrolateral PFC, dACC, and SMA in the HC group (Fig. 2 and Table 2). On the other hand, BD patients did not show this activation pattern (with the exception of left anterior insula in the very unfair > fair contrast). The opposite contrasts (i.e. fair > very unfair and fair > moderately unfair) revealed increased activation in the bilateral middle insula and right posterior insula regions only in the BD group (Fig. 2 and Table 2). In the control group, the typical reward-related activation of vmPFC in response to fair offers was observed.

**Table 2:** Within- and between-group brain activation in all contrasts.

	Cluster size	x^a^	y	z	T^b^
Moderately unfair > Fair offers					
HC group					
Dorsal anterior cingulate cortex / pre-SMA*	426	4	22	42	4.76
Left anterior insula*	86	–32	22	−6	4.14
Right anterior insula*	118	36	20	−6	3.80

BD group					
Left middle insula*	679	−46	−4	10	−6.02
Right posterior insula*	206	50	−24	30	−5.25
Right middle insula*	288	44	−2	10	−4.93

HC > BD					
Left middle insula*	411	−52	−4	10	4.35
Right posterior insula	53	46	−20	32	3.56
Right middle insula*	118	42	−2	10	3.46
Right anterior insula	99	36	14	−2	3.25

Very unfair > Fair offers					

HC group					
Right anterior insula*	156	38	20	−12	5.29
Left anterior insula*	73	−36	20	−8	3.92
Dorsal anterior cingulate cortex / pre-SMA	48	4	26	32	3.41
Ventromedial PFC	34	2	46	−14	−3.26

BD group					
Left anterior insula / ventrolateral PFC*	183	−38	20	−12	5.39
Left middle insula*	731	−46	−6	8	−6.12
Right posterior insula*	273	44	−28	32	−4.98
Right middle insula*	488	48	2	2	−4.82

Rejected > Accepted offers					

HC group					
Ventromedial PFC*	107	2	48	−6	−3.73

BD group					
Left ventrolateral PFC / Left anterior insula*	394	−38	22	−10	6.20
Right ventrolateral PFC / Right anterior insula	49	34	18	−20	3.43
Dorsal anterior cingulate cortex	112	2	26	34	3.27
Right middle insula*	300	52	2	18	−4.88
Left middle insula*	193	−46	−6	10	−4.68

HC > BD					
Dorsal anterior cingulate cortex*	48	2	12	22	−4.02
Left ventrolateral PFC / Left anterior insula*	117	−46	18	−12	−3.72

a Coordinates (x, y, z) reported in MNI space;

b All results significant at p < 0.05 cluster extent corrected across the set of predefined ROIs (p uncorrected < 0.005). Cluster size measured in voxels. Negative T values represent cluster that survived the opposite contrasts.

* Clusters that survived a cluster extent corrected across the set of predefined ROIs with a p uncorrected < 0.001.

**Fig. 3:**
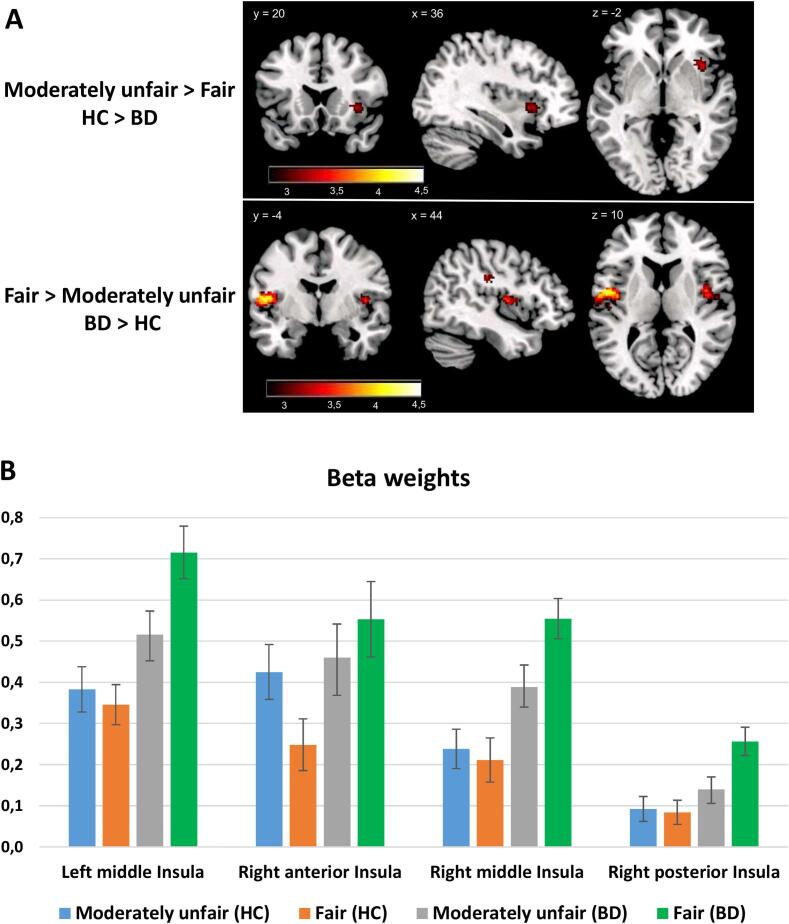
Between-group differences in neural responses to moderately unfair vs fair offers. A: The upper part depicts brain areas that display abnormal hypoactivation in the BD group for the Moderately unfair > Fair contrast. The lower part depicts brain areas that display stronger deactivation in the BD group for the Moderately unfair > Fair contrast. Images displayed at p < 0.005 initial threshold with a cluster extent threshold of 23 voxels. B: Average beta weights separately for the moderately unfair and fair offers in the four ROIs that displayed significant differences between HC (blue) and BD (orange). (For interpretation of the references to colour in this figure legend, the reader is referred to the web version of this article.)

The between-groups analysis revealed differences only in the moderately unfair vs fair contrast (Fig. 3 and Table 2). More specifically, moderately unfair offers elicited right anterior insula responses only in the HC group. Although this area is strongly implicated in the processing of unfairness, BD patients do not recruit this area in response to moderately unfair offers. On the other hand, the BD group displayed strong deactivation of bilateral middle insula and right posterior insula in response to moderately unfair offers. Healthy individuals did not exhibit deactivations in these areas in response to moderately unfair offers.

**Fig. 4:**
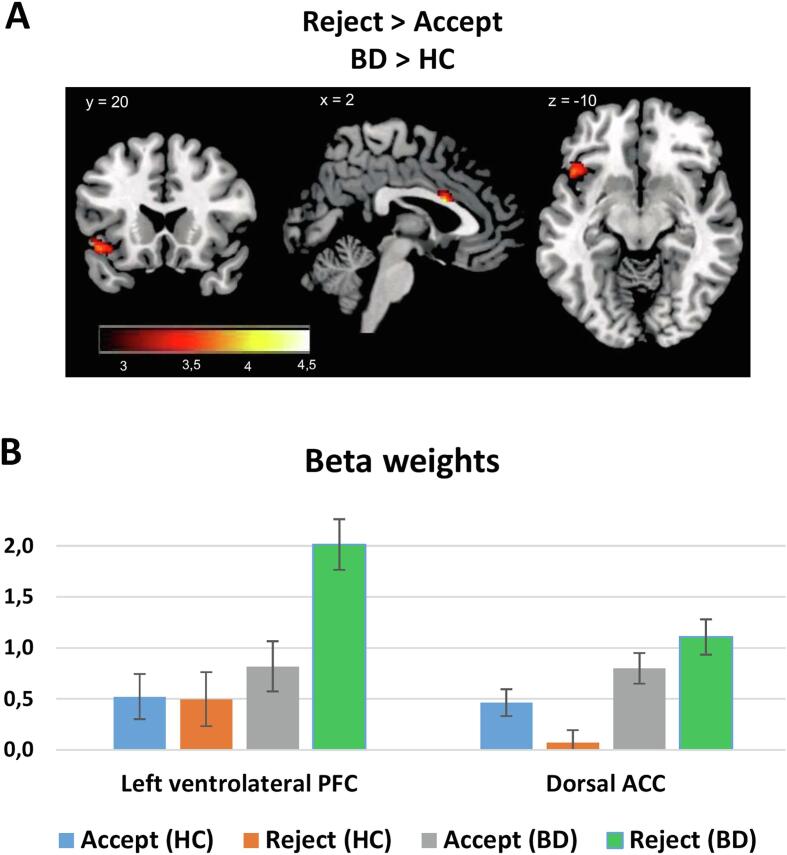
Between-group differences in neural responses to Rejected vs Accepted offers. A: The figure depicts brain areas that display abnormal hyperactivation in the Reject > Accept contrast. Images displayed at p < 0.005 initial threshold with a cluster extent threshold of 23 voxels. B: Average beta weights separately for the rejected and the accepted offers in the two ROIs that displayed significant differences between HC (blue) and BD (orange). (For interpretation of the references to colour in this figure legend, the reader is referred to the web version of this article.)

Comparing rejection vs acceptance of offers after the response prompt revealed distinct brain activation patterns in the two groups (Table 2). In the HC group, accepting vs rejecting an offer led to increased activation in the vmPFC. On the other hand, BD patients displayed increased acceptance-related activation in bilateral middle insula. Moreover, BD patients showed increased rejection-related activation in bilateral anterior insula/ventrolateral PFC and dorsal ACC. Direct comparison of the two groups revealed increased rejection-related activation in the left ventrolateral PFC and dorsal ACC in BD patients compared to HC (Fig. 4). Controlling for sociodemographic variables (i.e. age, gender, education, and income) did not influence the observed effects on brain activation.

### 3.4 Correlations with clinical variables, impulsivity, and affect intensity

Within the BD group, the voxel-wise regression analysis in the defined set of ROIs revealed a positive correlation between the level of impulsivity -measured by BIS11- and the extent of deactivation in response to moderately unfair offers in a cluster within the right posterior insula [(50, −20, 28), t = 4.56, n = 21]. The nonplanning factor of BIS11 seems to mainly drive this effect as it exhibited a positive correlation with deactivation in response to moderately unfair offers in a cluster within the same area of the right posterior insula [(50, −20, 28), t = 4.76, n = 31]. We observed no significant correlation between brain activation and BIS 11 within the HC group and no significant correlation between brain activation and affect intensity within the BD or the HC group.

The mediation analysis revealed that the 95% confidence interval representing the indirect effect of group (BD vs HC) on right posterior insula deactivation through impulsivity (BIS 11) did not include zero (0.0676 to 0.0010). Given that the direct effect of group on right posterior insula activation is still significant (0.1691 to 0.0147), the strong right posterior insula deactivation in BD patients seems to be partially mediated by their increased levels of impulsivity. No other cluster of BD-related abnormal activation is mediated by impulsivity or affect intensity.

We observed no correlation of abnormal brain activity in the BD group with depressive (HAMD) and manic (YMRS) residual symptoms or with any clinical variable. Moreover, the correlation analysis revealed no confounding effect of medication load (i.e. total and type-specific medication load) on the BD-related abnormal brain activity (see Table S3 in Supplementary Material).

## 4 Discussion

The present study is, to our knowledge, the first to investigate the neural basis of fairness perception in euthymic BD patients using the Ultimatum Game. At the behavioral level, we observed a decreased acceptance rate of moderately unfair offers in BD compared to HC. This behavioral aberration co-occurred with abnormal neural responses to moderately unfair offers in BD patients. More precisely, they exhibited hypoactivation of right anterior insula and showed stronger deactivation of posterior and middle insula in response to moderately unfair as compared to fair offers. A separate analysis that focused on the behavioral responses to the offers revealed a heightened rejection-related activation of dorsal ACC and left ventrolateral PFC in BD patients compared to HC.

Consistent with the single previous UG study in BD (Duek et al., 2014), we observed increased rejection rate of moderately unfair offers in BD patients. In addition, BD patients failed to demonstrate longer RTs to moderately unfair compared to fair or very unfair offers, a pattern that is typically observed in healthy individuals (Ferguson et al., 2014) and reflects increased cognitive effort during processing of moderately unfair offers. Taken together, these behavioral findings suggest that BD patients impulsively reject moderately unfair offers perceiving them as unfair. This interpretation is consistent with self-report measures showing that BD patients were more likely to express regret at behaving impulsively and rejecting offers (Duek et al., 2014).

At the brain level, healthy individuals displayed the typical unfairness-related activation in dorsal ACC/pre-SMA and bilateral anterior insula in response to very unfair and moderately unfair offers that many previous studies have reported (Feng et al., 2015, Gabay et al., 2014, Corradi-Dell'Acqua et al., 2013). This network of areas was not engaged during the presentation of very unfair and moderately unfair offers in BD. However, ventral portions of the dorsal ACC and bilateral anterior insula (including a large part of the ventrolateral PFC) were activated when BD patients rejected very unfair or moderately unfair offers. This pattern of results indicates an interesting dissociation between unfairness-driven brain activation and response-driven activation that warrants further investigation.

Previous studies have implicated the dorsal ACC in the monitoring of motivational conflict that responders face in the UG between economic self-interest and intuitive emotion-driven responses to fairness violations (Botvinick et al., 2004, Civai et al., 2012, Xiang et al., 2013, Zhou et al., 2014). Interestingly, when self-interest does not play a role in norm enforcement (i.e. third-party UG) and when individuals are not prone to self-interest (Civai et al., 2012), dorsal ACC is not engaged. BD patients activated a ventral portion of this area only after the response prompt and only when the offers were rejected. Therefore, BD patients seem to experience this motivational conflict when they reject an unfair offer and not when the unfair offer is presented, as healthy individuals do. One interesting question for future research is whether the increased dorsal ACC activation during offer rejection can predict the subsequent feelings of regret that some BD patients reported (Duek et al., 2014).

Several studies have implicated the anterior insula in the visceral experience of negative feelings related to unfairness (Sanfey et al., 2003, Tabibnia and Lieberman, 2007, Sanfey and Chang, 2008, Grecucci et al., 2013, Harle et al., 2012). Some of these studies have shown that emotion infusion (Harle et al., 2012) and emotion regulation (Grecucci et al., 2013) modulate the unfairness‐evoked AI responses, which predict behavioral responses (Sanfey et al., 2003, Tabibnia and Lieberman, 2007, Harle et al., 2012). BD patients did not recruit bilateral anterior insular regions during the processing of very unfair or moderately unfair offers and the direct comparison of the two groups revealed an abnormal hypoactivation of right anterior insula in response to moderately unfair offers. On the other hand, the reject vs accept contrast revealed an abnormal hyperactivation of left ventrolateral PFC/left anterior insula in BD.

Recent neuroimaging evidence can offer a plausible explanation of this pattern of results based on a functional heterogeneity among different anterior insular subregions and adjacent PFC regions (Zhou et al., 2014, Chang et al., 2013, Kelly et al., 2012). According to this view, the ventral anterior insula and adjacent ventrolateral PFC have been linked to processing of emotions while the dorsal parts of the anterior insula is associated with cognitive functions and is more responsive to contextual cues (Zhou et al., 2014, Chang et al., 2013). Therefore, it has been argued that anterior insula plays a major role in integrating interoceptive information with cognitive appraisals and contextual information in order to promote socially appropriate behavior (Corradi-Dell'Acqua et al., 2013, Janiri et al., 2019). In this respect, the hypoactivation of right anterior insula in response to moderately unfair offers may reflect an impaired integration of contextual information in BD that may result in an emotion-driven rejection of moderately unfair offers. In line with this view, the cognitive deficits that characterize mood and anxiety disorders in general have been strongly linked to hypoactivation of right inferior prefrontal/insular cortex during cognitive control tasks (McTeague et al., 2017). Within the same framework, the rejection-related abnormally increased activation in left ventral AI and ventrolateral PFC may reflect increased emotional resentment of BD patients that leads to offer rejection. This explanation resonates with a previous UG study in which BD patients experienced increased anger after receiving unfair offers compared to healthy controls (Duek et al., 2014).

The BD group demonstrated a strong deactivation of posterior and middle insula in response to moderately unfair offers. This deactivation was absent in healthy individuals. Previous UG studies have implicated the posterior and middle insula in the encoding of fairness and equality (Hsu et al., 2008, Wright et al., 2011). In an effort to disentangle the objective from the contextual aspects of fairness, Wright and his colleagues (2011) showed that posterior and middle insula are involved in the construction of fairness perception that adapts to social contextual cues (Wright et al., 2011). In light of this finding, the deactivation of posterior and middle insula in response to moderately unfair offers may reflect the failure of BD patients to integrate contextual information during the processing of these offers. Within this framework, it can be speculated that BD patients focus only on the absolute inequality of moderately unfair offers and impulsively respond as pure egalitarians. Therefore, the failure of BD patients to integrate contextual information may reflect the tendency of BD patients to impulsively respond to stimuli without engaging in higher cognitive level processing (Etain et al., 2013, Swann et al., 2003). Importantly, this explanation is in line with the fact that trait impulsivity partially mediated the group effect on the deactivation of right posterior insula in moderately unfair offers. Furthermore, this explanation fits the RT pattern where, unlike HC, BD patients do not seem to process moderately unfair offers longer than the other types of offers.

Nevertheless, this interpretation should be considered with caution. Previous work has shown that healthy individuals display a deactivation of middle and posterior insula in response to unfair vs fair offers (Feng et al., 2015). In the present study, this insula deactivation was present in the patient group but not in the HC group. In this respect, an alternative interpretation of the present findings is that healthy individuals did not exhibit the expected deactivation of middle and posterior insula in response to unfairness. However, this alternative interpretation is less plausible since the patient group (compared to the control group) displayed significantly stronger deactivation only in response to moderately unfair offers (i.e. offers which under the present highly unfair context may be perceived as relatively fair).

The hypothesized inability to integrate contextual information is also consistent with evidence showing dysfunctional integration of contextual cues in BD during emotion recognition and empathy tasks (Baez et al., 2013) as well as cognitive control tasks (Brambilla et al., 2007). In one of these studies, the authors argued that social cognition deficits in ambiguous situations could be explained by patient’s inability to use contextual cues to infer the actor’s intentionality (Baez et al., 2013). In the case of the UG, healthy individuals may perceive a moderately unfair offer as an effort from the proposer to balance his/her self-interest motives with his/her intention to be fair. BD patients, on the other hand, may fail to consider proposer’s intentions resulting in increased rejection rates of moderately unfair offers and heightened experienced anger (Duek et al., 2014). Despite its plausibility, this assumption should be considered with caution as no data on the perceived intentions of the proposer were collected.

One important limitation of the present study is the use of a liberal voxel-based threshold (p_uncor_ < 0.005) for cluster-extent based thresholding. This decision was based on the fact that the present study is the first to investigate the neural basis of fairness in BD and thus our findings are more exploratory. Nevertheless, using a more commonly used voxel-based threshold (p_uncor_ < 0.001) resulted in a similar brain activation pattern (see Table S2 in Supplementary Material). However, two between-group clusters (right anterior insula and right posterior insula) did not survive this more stringent threshold. Given the important role of these two insula regions in processing fairness, future studies are warranted to examine the robustness of these findings in the BD population and in populations at risk for developing affective disorders.

Similar to previous studies that used the UG (Sanfey et al., 2003, Corradi-Dell'Acqua et al., 2013, Civai et al., 2012), in the present study, the material value of each offer is positively correlated with the fairness of the offer. This constitutes an important limitation that may confound the fairness effect on behavior and brain activation with a potential material value effect. However, a previous UG study (Zhou et al., 2014) that systematically addressed this issue showed no main effect of stake size on brain activation and a significant fairness by stake size interaction in which fairness-related brain activation was more pronounced when the stakes were high. Moreover, assuming an effect of stake size on behavior, we would expect a fixed marginal increase in acceptance rates in response to a fixed marginal increase in the size of the stake (e.g. adding on 1€). However, results indicate that increasing the offer from 1€ to 2€ leads to only a 10% increase in acceptance rates while increasing the offer from 2€ to 3€ (again a 1€ absolute increase in stake size) leads to a 30% increase in acceptance rates. This pattern of results follows the typical acceptance rates by fairness level interaction that is observed in other UG studies (Gabay et al., 2014) suggesting that participants paid more attention to the fairness than the absolute material value of the offers.

The homogeneity of our clinical sample may limit the interpretation of the present findings. BD is characterized by increased prevalence of comorbid disorders. However, in our sample of euthymic BD patients current or past comorbidities were rather rare. Extending this argument, future studies are warranted not only to recruit a more representative sample of euthymic BD patients, but also to examine how symptomatic patients or populations at risk for BD respond to perceived unfairness in social settings.

### 4.1 Conclusions

Despite these limitations, the present findings provide important insights into the neurocognitive mechanisms underlying the processing of fairness perception in BD and highlight the potential crucial role of ambiguity and contextual processing in the manifestation of BD social deficits. Given the ubiquity of ambiguous social situations in everyday life, we argue that the social deficits that characterize BD patients may manifest predominantly during situations when context integration is crucial.

## CRediT authorship contribution statement

**Giannis Lois:** Conceptualization, Methodology, Formal analysis, Investigation, Writing - original draft. **Eva E. Schneider:** Investigation, Writing - review & editing. **Aleksandra Kaurin:** Investigation, Writing - review & editing. **Michèle Wessa:** Writing - review & editing, Funding acquisition, Project administration.

## Declaration of Competing Interest

The authors declare that they have no known competing financial interests or personal relationships that could have appeared to influence the work reported in this paper.
