# Supplementary material for: Altered neural responses to social fairness in bipolar disorder
Source: Neuroimage Clin. 2020 Nov 3;28:102487. doi: 10.1016/j.nicl.2020.102487 (PMC7666350; doi:10.1016/j.nicl.2020.102487)
Supplement: Supplementary Data 1 [file mmc1.docx]

**Supplemental Material and Results**

pertaining to the study:

**Altered neural responses to social fairness in Bipolar Disorder**

Giannis Lois^a,b^, Eva E. Schneider^a^, Aleksandra Kaurin^a, c, d^, Michèle Wessa^a, c^

1. **Materials and methods**

**
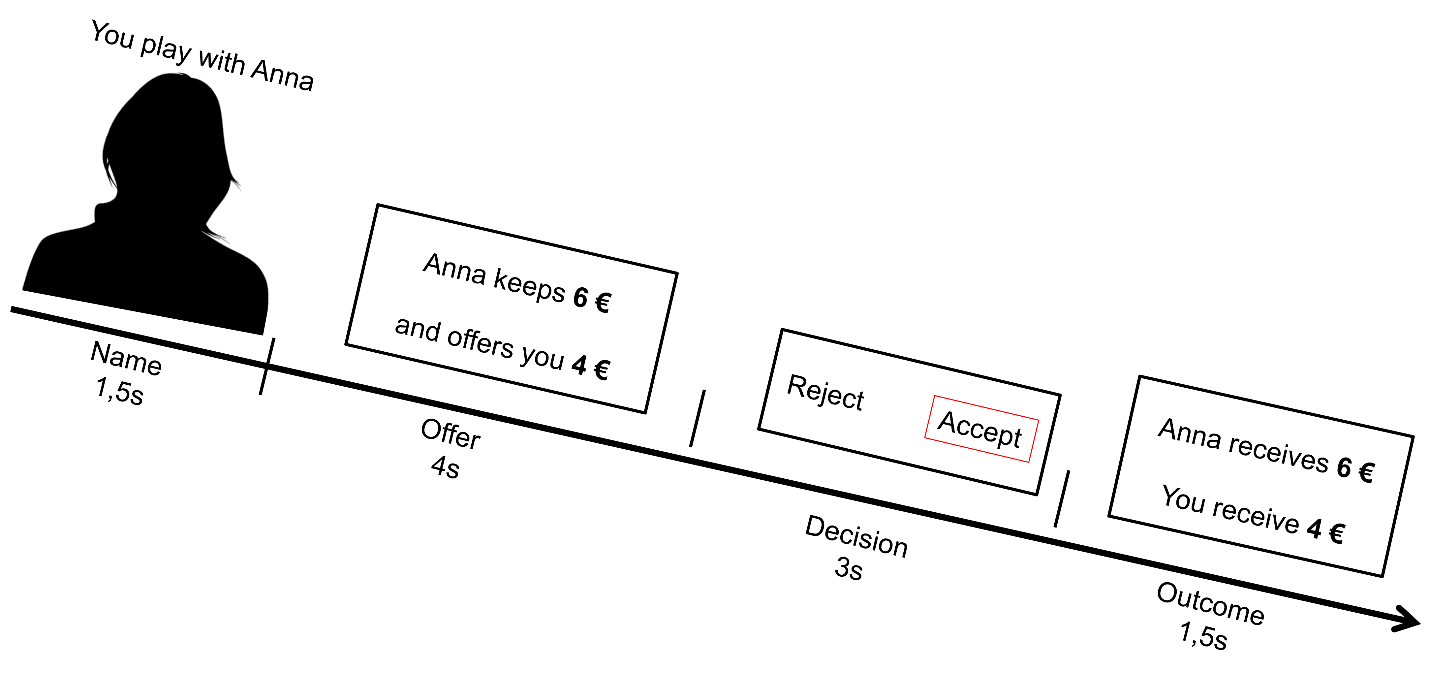
**

Figure S1. Sequence and timing of a typical trial of the Ultimatum Game used in the present study

**2. Results**

**Table S1.** Within- and between-group whole brain activation in all contrasts

|  | **Cluster size** | **x^a^** | **y** | **z** | **T^b^** |
| --- | --- | --- | --- | --- | --- |
| **Moderately unfair > Fair offers** |  |  |  |  |  |
| **HC group** |  |  |  |  |  |
| Dorsal anterior cingulate cortex / pre-SMA | 350 | 4 | 22 | 42 | 4.76 |
| Left anterior insula | 33 | -32 | 22 | -6 | 4.14 |
| Right anterior insula | 26 | 36 | 20 | -6 | 3.80 |
| Left inferior parietal gyrus | 159 | -58 | -44 | 46 | -4.69 |
| Left middle occipital gyrus | 112 | -44 | -78 | 36 | -4.67 |
| Posterior cingulate cortex | 112 | 8 | -40 | 38 | -4.33 |
| **BD group** |  |  |  |  |  |
| Left middle insula | 1813 | -46 | -4 | 10 | -6.02 |
| Right posterior insula | 1570 | 50 | -32 | 30 | -6.72 |
| Left precuneus | 1432 | -14 | -48 | 62 | -5.69 |
| SMA | 1058 | -6 | -8 | 54 | -5.48 |
| Right postcentral gyrus | 618 | 24 | -46 | 62 | -5.08 |
| **HC > BD** |  |  |  |  |  |
| Left middle insula | 312 | -52 | -4 | 10 | 4.35 |
| Dorsal anterior cingulate cortex | 48 | -8 | 8 | 36 | 4.15 |
| Right posterior insula | 106 | 38 | -10 | 38 | 4.08 |
| Right middle insula | 26 | 42 | -2 | 10 | 3.46 |
| SMA | 38 | 10 | -4 | 56 | 3.77 |
| Right precentral gyrus | 95 | 56 | 0 | 38 | 3.98 |
| **Very unfair > Fair offers** |  |  |  |  |  |
| **HC group** |  |  |  |  |  |
| Right anterior insula | 95 | 38 | 20 | -12 | 5.29 |
| Left anterior insula | 53 | -34 | 18 | -8 | 3.99 |
| Dorsal anterior cingulate cortex / pre-SMA | 48 | 4 | 26 | 32 | 3.41 |
| Left middle frontal gyrus | 519 | -40 | 38 | 20 | -6.08 |
| Posterior cingulate cortex | 1737 | -8 | -34 | 38 | -5.93 |
| Left middle temporal gyrus | 194 | -62 | -48 | -10 | -5.15 |
| Right middle temporal gyrus | 180 | 56 | -44 | -6 | -4.68 |
| Left middle occipital gyrus | 313 | -44 | -78 | 36 | -4.50 |
| Left dorsolateral PFC | 186 | -26 | 20 | 48 | -4.46 |
| Ventromedial PFC | 15 | 2 | 46 | -14 | -3.26 |
| **BD group** |  |  |  |  |  |
| Left anterior insula / ventrolateral PFC | 159 | -38 | 20 | -12 | 5.39 |
| Left pre-SMA | 49 | -10 | 14 | 66 | 4.47 |
| Right pre-SMA | 110 | 10 | 18 | 68 | 4.31 |
| Anterior medial PFC | 155 | -2 | 48 | 24 | 4.03 |
| Left middle insula | 1245 | -46 | -6 | 8 | -6.12 |
| Posterior cingulate cortex | 1254 | -10 | -28 | 40 | -6.87 |
| Left inferior temporal gyrus | 307 | -60 | -46 | -14 | -5.97 |
| Cuneues and medial occipital gyrus | 4273 | -14 | -74 | 26 | -5.56 |
| Left precentral gyrus | 536 | -54 | -4 | 36 | -5.54 |
| Right fusiform gyrus | 371 | 30 | -42 | -12 | -5.37 |

**Table S1 to be continued**

| **Rejected > Accepted offers** |  |  |  |  |  |
| --- | --- | --- | --- | --- | --- |
| **HC group** |  |  |  |  |  |
| Cerebellum | 157 | 16 | -40 | -14 | -5.88 |
| Posterior cingulate cortex | 761 | 6 | -36 | 38 | -5.65 |
| Left inferior temporal gyrus | 155 | 58 | -42 | -10 | -5.31 |
| Right angular gyrus | 689 | 46 | -70 | 32 | -4.93 |
| Ventromedial PFC | 122 | 2 | 48 | -6 | -3.73 |
| **BD group** |  |  |  |  |  |
| Left ventrolateral PFC / Left anterior insula | 544 | -36 | 20 | -14 | 6.23 |
| Left pre-SMA | 148 | -10 | 14 | 66 | 4.79 |
| Dorsal anterior cingulate cortex | 590 | 2 | 48 | 36 | 4.63 |
| Right pre-SMA | 120 | 10 | 18 | 60 | 4.34 |
| Right middle insula | 311 | 56 | 4 | 18 | -5.45 |
| Left middle insula | 90 | -46 | -6 | 10 | -4.68 |
| Left inferior orbitofrontal gyrus | 124 | -20 | 28 | -10 | -4.62 |
| Right postcentral gyrus | 58 | 28 | -32 | 42 | -4.48 |
| Left inferior temoral gyrus | 62 | -58 | -48 | -14 | -4.47 |
| **HC > BD** |  |  |  |  |  |
| Dorsal anterior cingulate cortex | 95 | 0 | 12 | 22 | -4.33 |
| Cerebellum | 97 | 4 | -52 | -38 | -4.27 |
| Right anterior lateral PFC | 34 | 20 | 34 | 38 | -3.91 |
| Right dorsolateral PFC | 50 | 42 | 10 | 52 | -3.87 |
| Left ventrolateral PFC / Left anterior insula | 49 | -46 | 18 | -12 | -3.72 |

^a^ Coordinates (x. y. z) reported in MNI space;

^b^ All results significant at p<0.05 cluster extent corrected across the set of predefined ROIs (p uncorrected <0.005). Cluster size measured in voxels. Negative T values represent cluster that survived the opposite contrasts.

**Table S2.** Within- and between-group activation in all contrasts within the defined set of ROIs

|  | **Cluster size** | **x^a^** | **y** | **z** | **T^b^** | **p_FWE_ peak** | **p_FWE_ cluster** |
| --- | --- | --- | --- | --- | --- | --- | --- |
| **Moderately unfair > Fair offers** |  |  |  |  |  |  |  |
| **HC group** |  |  |  |  |  |  |  |
| Dorsal anterior cingulate cortex / pre-SMA | 255 | 4 | 22 | 42 | 4.76 | .040 | .059 |
| Left anterior insula | 20 | -32 | 22 | -6 | 4.14 | .052 | .129 |
| Right anterior insula | 26 | 36 | 20 | -6 | 3.80 | .117 | .087 |
| **BD group** |  |  |  |  |  |  |  |
| Left middle insula | 399 | -46 | -4 | 10 | -6.02 | <.001 | <.001 |
| Right posterior insula | 139 | 50 | -24 | 30 | -5.25 | .003 | .003 |
| Right middle insula | 166 | 44 | -2 | 10 | -4.93 | .004 | .002 |
| **HC > BD** |  |  |  |  |  |  |  |
| Left middle insula | 186 | -52 | -4 | 10 | 4.35 | .014 | .004 |
| Right posterior insula | 6 | 46 | -20 | 32 | 3.72 | .091 | .196 |
| Right middle insula | 19 | 42 | -2 | 10 | 3.46 | .122 | .110 |
| **Very unfair > Fair offers** |  |  |  |  |  |  |  |
| **HC group** |  |  |  |  |  |  |  |
| Right anterior insula | 82 | 38 | 20 | -12 | 5.29 | .008 | .022 |
| Left anterior insula | 23 | -36 | 20 | -8 | 3.92 | .121 | .128 |
| **BD group** |  |  |  |  |  |  |  |
| Left anterior insula / ventrolateral PFC | 84 | -38 | 20 | -12 | 5.39 | .003 | .016 |
| Left middle insula | 476 | -46 | -6 | 8 | -6.12 | <.001 | <.001 |
| Right posterior insula | 176 | 44 | -28 | 32 | -4.98 | <.001 | .001 |
| Right middle insula | 420 | 48 | 2 | 2 | -4.82 | .001 | <.001 |
| **Rejected > Accepted offers** |  |  |  |  |  |  |  |
| **HC group** |  |  |  |  |  |  |  |
| Ventromedial PFC | 21 | 2 | 48 | -6 | -3.73 | .255 | .164 |
| **BD group** |  |  |  |  |  |  |  |
| Left ventrolateral PFC / Left anterior insula | 288 | -38 | 22 | -10 | 6.20 | <.001 | <.001 |
| Right middle insula | 133 | 52 | 2 | 18 | -4.88 | .011 | .003 |
| Left middle insula | 86 | -46 | -6 | 10 | -4.68 | .017 | .010 |
| **HC > BD** |  |  |  |  |  |  |  |
| Dorsal anterior cingulate cortex | 18 | 2 | 12 | 22 | -4.02 | .033 | .082 |
| Left ventrolateral PFC / Left anterior insula | 43 | -46 | 18 | -12 | -3.72 | .073 | .032 |

^a^ Coordinates (x. y. z) reported in MNI space;

^b^ All results significant at p<0.05 cluster extent corrected across the set of predefined ROIs (p uncorrected <0.001). Cluster size measured in voxels. Negative T values represent cluster that survived the opposite contrasts.

**Table S3.** Pearson correlations between clinical characteristics (vertical axis) and brain activation in ROIs that displayed group differences (horizontal axis) separately for each group.

|  | **Healthy control group** | | | | | | **Bipolar disorder group** | | | | | |
| --- | --- | --- | --- | --- | --- | --- | --- | --- | --- | --- | --- | --- |
|  | **Left middle insula** | **Right anterior insula** | **Right middle insula** | **Right posterior insula** | **Dorsal ACC** | **Left ventrolateral PFC** | **Left middle insula** | **Right anterior insula** | **Right middle insula** | **Right posterior insula** | **Dorsal ACC** | **Left ventrolateral PFC** |
| **YMRS** | -.028 | .046 | -.004 | -.172 | .010 | .044 | .114 | .100 | .257 | .188 | -.186 | -.123 |
| **HAMD** | .182 | .110 | .137 | .117 | -.314 | -.306 | .015 | -.119 | .057 | -.230 | -.097 | .114 |
| **No. of depressive episodes** | - | - | - | - | - | - | .140 | -.257 | .104 | .227 | .306 | -.130 |
| **No of manic episodes** | - | - | - | - | - | - | .192 | -.156 | .177 | .193 | .310 | -.256 |
| **Age at illness onset**  **(years)** | - | - | - | - | - | - | .112 | -.022 | .163 | .156 | .147 | .214 |
| **Time in remission (months)** | - | - | - | - | - | - | -.190 | -.072 | -.036 | -.034 | -.150 | -.096 |
| **Total med load** | - | - | - | - | - | - | -.014 | -.264 | -.152 | -.031 | -.044 | .075 |
| **SSRI antidepressants med load** | - | - | - | - | - | - | -.026 | -.192 | .013 | .023 | .127 | .076 |
| **Other antidepressants med load** | - | - | - | - | - | - | .075 | .029 | .040 | .025 | -.088 | -.166 |
| **Mood stabilizers med load** | - | - | - | - | - | - | .226 | .171 | .049 | -.024 | -.320 | .001 |
| **Antipsychotics med load** | - | - | - | - | - | - | .021 | -.067 | -.141 | -.026 | -.012 | .134 |
|  |  |  |  |  |  |  |  |  |  |  |  |  |
